# Characteristics of HIV-infected women and factors associated with HCV seropositivity in the Republic of Georgia

**DOI:** 10.1186/1742-6405-8-25

**Published:** 2011-07-25

**Authors:** Nikoloz Chkhartishvili, Louise-Anne McNutt, Perry F Smith, Tengiz Tsertsvadze

**Affiliations:** 1 Infectious Diseases, AIDS and Clinical Immunology Research Center, 16 Al. Kazbegi Avenue, Tbilisi 0160, Georgia; 2 Department of Epidemiology and Biostatistics, University at Albany School of Public Health, One University Place Rensselaer, NY 12144, USA; 3 Department of Epidemiology, New York State Department of Health, ESP Corning Tower, Room 503, Albany, NY 12237, USA; 4 Tbilisi State University Faculty of Medicine, 16 Al. Kazbegi Avenue, Tbilisi 0160, Georgia

## Abstract

**Background:**

The aim of this study was to describe the extent of the HIV epidemic among women in the Republic of Georgia and to identify factors associated with HCV co-infection in this population.

**Findings:**

All women aged ≥18 years who were diagnosed with HIV between 1989 and 2006 were identified through the National HIV/AIDS surveillance database. Medical records were reviewed for demographic characteristics, risk factors and HCV serostatus. A total of 249 women were identified. Only 4% declared injection drug use (IDU); sex work was reported by 9%. Substantial risk factors were identified among the women's sexual partners, nearly 69% of whom were IDUs, 84% were HIV positive and 66% HCV positive. Seventeen percent of women were seropositive for HCV. Factors significantly associated with HCV seropositivity in bivariate analyses among non-IDU women were partner IDU+ [Prevalence ratio (PR): 4.5 (95% CI: 1.4, 14.2)], and partner HCV+ [PR: 7.2 (95% CI: 1.8, 29.5)].

**Conclusions:**

The HIV epidemic in the Republic of Georgia is closely tied to the IDU community. Evidence-based interventions targeting IDU and partners of IDU are urgently required to halt the spread of the HIV epidemic in the country.

## Findings

The Newly Independent States (NIS) of Eurasia have the highest rates of HIV infection in the region [1,2]. The HIV epidemic here has been closely linked with the socioeconomic and political upheavals of the early 1990s and the collapse of the Soviet Union. The emergence of drug markets and injection drug use (IDU) is now the major driver of the epidemic in the region [3,4]. The burgeoning IDU population was accompanied by an explosive spread of hepatitis C virus (HCV) and HIV; today, the IDU population is almost universally infected with HCV and the majority is co-infected with HIV [5-7].

**Figure 1:**
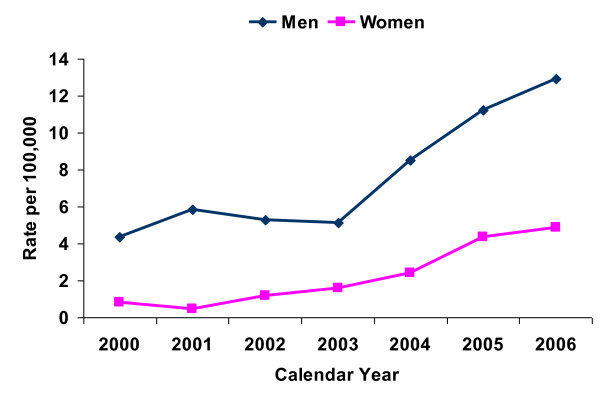
**HIV incidence among adult men and women in the Republic of Georgia, 2000-2006**.

Formerly part of the Soviet Union, Georgia is an independent nation with a population of about 4.5 million. Compared to other NIS, Georgia's HIV epidemic started later and grew more slowly. The first case of HIV was reported in 1989; as of December 31, 2006, a cumulative 1,156 HIV cases had been reported. Nearly 78% of cases are men and 22% women. Although the estimated HIV prevalence in Georgia is low (< 0.1%), the rate of new infections has risen each year (see Figure 1). Among men, approximately 80% of cases are attributable to IDU. The vast majority of HIV positive women (93%) were exposed through heterosexual contact.

Similar to other countries in the region, the spread of HCV infection in Georgia was partially associated with the rise in IDU. Multiple studies have found strong associations between male gender, IDU and HCV infection. While HIV prevalence in IDUs is relatively low (2.2%), HCV infection is common (70%) [8-11]. In these studies IDUs almost exclusively were male. Little is known about the HCV transmission risks among HIV-positive women. This study describes the HIV epidemic among women and identifies risk factors associated with HCV seropositivity among HIV positive women.

## Methods

The study was conducted at the Infectious Diseases, AIDS & Clinical Immunology Research Center (IDACIRC), which is Georgia's referral institution for HIV diagnosis, treatment and care. IDACIRC is the country's sole institution providing confirmation testing of initially positive HIV cases. All individuals with a confirmed HIV diagnosis are first seen at IDACIRC in Tbilisi. In addition to clinical assessment the initial exam includes information on socio-demographic characteristics and risk factors. Data are subsequently entered into HIV/AIDS electronic database operated by the IDACIRC. The database contains information on all individuals with confirmed HIV infection since the first case reported in 1989.

The IDACIRC also coordinates treatment for individuals infected by HIV. Treatment for HIV/AIDS in Tbilisi, the nation's capital, has been provided at the IDACIRC since the beginning of the epidemic. In 2006, two regional AIDS treatment facilities were opened, providing local treatment options for individuals living outside Tbilisi after initial assessment.

### Study Population

We included all women at least 18 years of age diagnosed with HIV between 1989 and 2006 who were identified and received evaluation and treatment at the Tbilisi IDACIRC office.

### Data

The database contains person's name, demographic characteristics, medical identification, date of HIV diagnosis, disease stage, and exposure risk category, among other factors. Medical records at the IDACIRC were reviewed for demographic characteristics, risk factors and HCV serostatus.

### Factors

*Demographic characteristics *abstracted included age, ethnicity, educational level, employment, marital status, residence and refugee (internally displaced) status.

*Risk factors for HIV *are ascertained during patients' initial examinations with detailed questions regarding IDU, sexual activity, history of sexually transmitted infection (STI), viral hepatitis, commercial sex work, risk factors of the patient's last regular sexual partner(s), and medical care-related risk factors (e.g., history of invasive medical manipulations and blood transfusion). Information on patient's main sexual partner was extracted from the women's detailed intake exam records; additionally partners' medical records were reviewed for those women with HIV positive partners registered at the IDACIRC.

*Screening for HCV infection *is routinely performed among persons with HIV. Co-infection is defined as the presence of HCV antibodies detected by second- and third-generation enzyme immunoassay and documented in the medical record [12].

### Statistical Analysis

All statistical analyses were performed using SAS v. 9.1.3. Descriptive statistics were used to examine variable distributions. Bivariate associations between risk factors and HCV co-infection were assessed using prevalence ratios (PR) and their 95% confidence intervals (CI). Multivariate analyses by stratified analysis and Poisson regression with robust variance estimates were utilized to assess associations between multiple covariates and HCV co-infection [13,14].

### Ethical Approval

Study was approved by the Institutional Review Boards (IRB) of the IDACIRC and University at Albany.

## Results

A total of 249 women aged 18 years or older with a confirmed HIV diagnosis were identified from the HIV/AIDS surveillance database. HCV status was available for 244 women. Because of substantial missing data, analyses were generally limited to 228 women. Women with complete and incomplete data did not differ in terms of demographic characteristics or personal behaviors. The majority of missing data resulting in exclusion from analyses was partner-related risk factors.

To understand the extent of the HIV epidemic, we reviewed surveillance data for women by source of identification. Most of the women in our study were identified through HIV case investigation, self referral, prenatal screening or referral from an HIV lab. Of 78,776 pregnant women screened since the prenatal screening program began in 2003, 32 (0.0004) were HIV-positive, and of these, 21 (66%) reported partner-related risk factors: IDU, HIV+ and HCV + status. The other 11 were not known to have any IDU connection. Other important sources of identification included case investigation, referral from medical facilities and self-referral. The majority of these women had documented connection to IDUs (i.e., partner was IDU) ranging from 52% to 80%.

Among the 228 HIV+ women whose medical records were abstracted, 68% were younger than 35 years of age (mean age: 31 years). The vast majority were ethnic Georgians (89%), unemployed (76%) and married (68%) at the time of diagnosis. Only 32% were college graduates. Thirteen percent were internally displaced from occupied regions of Georgia.

Relatively few women reported major behavioral risk factors for HIV or HCV infection. Only ten (4%) women declared use of injection drugs; commercial sex work was reported by 21 (9%) women. While 36% of women had more than one lifetime sexual partner, overall more than half reported no personal risk behavior for HIV other than having a partner who is IDU. The sexual partners brought substantial risk factors to the relationship: the majority were HIV positive (84%), nearly 69% gave a history of IDU, and approximately 66% had documented HCV antibodies. About 30% of women had a history of an STI. Approximately half of the women reported a history of at least one invasive medical procedure.

Among the 10 IDU women, eight (80%) were positive for HCV antibodies, while among the 218 who did not report IDU, 32 (14.7%) women were HCV seropositive. Because of the strong association between IDU and HCV infection [PR: 5.4 (95% CI: 3.5-8.5)], IDU women were excluded from further analyses.

Factors significantly associated with HCV seropositivity in bivariate analyses among self-declared non-IDU women were IDU partner [PR: 4.5 (95% CI: 1.4-14.2)], and partner HCV seropositivity [PR: 7.2 (95% CI: 1.8-29.5)]. In a multivariate Poisson regression (with robust variance estimates), only partner HCV positivity remained significantly associated with the woman's HCV co-infection: PR 8.1 (95% CI: 1.9, 34.0) (Table 1). Assessment of the strongest bivariate risk factors for HCV-coinfection in a multivariate manner provided insight into the regression model's results (Table 2). A strong association existed between the partner's IDU-status and the woman's HCV seropositivity; however, due to substantial colinearity, it was the partner's HCV status that was the most predictive factor for women being infected with HCV.

**Table 1:** Association between risk factors and hepatitis C virus (HCV) co-infection among 218 self-declared non-injection drug using women with HIV in the Republic of Georgia

		HCV Positive		Bivariate analysis	Multivariate analysis*
		
	Total N	N	%	PR (95% CI)	PR (95% CI)
**Demographic Characteristics**					
Age					
18-34	150	25	16.7	1.6 (0.7, 3.6)	
≥35	68	7	10.3		
Education					
No degree	147	21	14.3	0.9 (0.5, 1.8)	
College/university degree	71	11	15.5		
Employment					
Unemployed	163	27	16.6	1.8 (0.7, 4.5)	
Employed	55	5	9.1		
Marital status					
Not married	57	8	14.0	0.9 (0.4, 1.9)	1.3 (0.6, 2.7)
Married	161	24	14.9		
Internally displaced					
Yes	29	4	13.8	0.9 (0.4, 2.5)	
No	189	28	14.8		
**Medical Care**					
History of surgery					
Yes	98	14	14.3	0.9 (0.5, 1.8)	0.8 (0.4, 1.7)
No	120	18	15.0		
History of STI					
Yes	59	8	13.6	0.9 (0.4, 1.9)	1.3 (0.6, 2.7)
No	159	24	15.1		
**Personal Risk Behaviors**					
Sex work					
Yes	14	1	7.1	0.5 (0.1, 3.2)	
No	204	31	15.2		
Number of sexual partners					
> 1 partner	70	10	14.3	0.9 (0.5, 1.9)	
1 partner	148	22	14.9		
**Partner Related Factors**					
Partner risk factor					
IDU documented	149	29	19.5	4.5 (1.4, 14.2)	
IDU not documented	69	3	4.4		
Partner HIV status					
HIV positive	185	31	16.8	5.5 (0.8, 39.1)	
HIV negative/no regular partner	33	1	3.0		
Partner HCV status					
HCV positive	147	30	20.4	7.2 (1.8, 29.5)	8.1 (1.9, 34.0)
HCV negative/no regular partner	71	2	2.8		

* Partner IDU status and partner HIV status were dropped from the model due to colinearity with partner's HCV status. Initial multivariate model included all variables. The final model retained variables "Marital status", "history of STI" and "Marital status" because of confounding effect.

**Table 2:** Stratified analysis between partner-related factors and women's hepatitis C virus (HCV) status among 244 HIV positive women with known HCV status in the Republic of Georgia

		HCV positive	
	Total N	N	%
Women, IDU*+	**10**	**8**	**80.0**
**Women, no IDU documented**	**199**	**32**	**14.7**
**Partner HCV+**	**147**	**30**	**20.4**
Partner IDU+	141	29	20.6
Partner no IDU documented	6	1	16.7
**Partner HCV-**	**52**	**1**	**1.9**
Partner IDU+	8	0	0
Partner no IDU documented	44	1	2.3
**No regular partner reported**	**19**	**1**	**5.3**
**Women with missing partner information**	**16**	**2**	**12.5**

IDU: injection drug use.

## Discussion

This study examined all known HIV positive women in Georgia and found that the HIV epidemic remains closely tied to the IDU community. The majority of HIV positive women (70%) have a documented connection to the IDU community through either their own IDU (n = 10) or a partner with IDU (n = 163) or HCV (a proxy for IDU among men in the region, n = 152).

While few women reported IDU, HCV in women is common for those with partners who are HCV seropositive. The fact that most women reported no risk behavior related to HCV does not necessarily mean that the infection was transmitted through heterosexual exposure. Although sexual transmission of HCV may occur [15,16], the overwhelming evidence suggests that the efficiency of transmission by the sexual route is very low. Longitudinal studies among monogamous heterosexual couples indicate no or low risk associated with acquisition of HCV through sexual intercourse. Vandelli and colleagues reported an incidence rate of 0.37/1,000 person-years (py) among monogamous heterosexual couples with over 10 years of follow-up [17]. In another follow-up study, incidence of HCV acquisition was estimated at 2.33/1,000py [18]. Two other studies reported an absence of seroconversion among spouses [19,20].

On the other hand, previous studies have shown that those with risky sexual behavior, STIs (particularly genital ulcerative diseases), and co-infection with HIV have an increased risk of HCV acquisition [21-26]. Consistent with these reports our study suggests that having sexual contact with HCV positive partners is an important co-factor for being HCV positive among HIV infected women in Georgia. However, it is unlikely that the high prevalence of HCV in our study was solely due to the sexual exposure to the virus.

Evidence suggests that IDU is one of the most efficient modes of HCV transmission,[27] which can occur shortly after initiation of IDU [28]. Within the ALIVE Study, 65% of participants with brief IDU were positive for HCV antibodies [29]. And HCV seroprevalence exceeding 70% was reported in a similar study in Southern China [30]. Cohort studies among persons with IDU showed that the incidence of HCV was greatest within the first year of follow-up and ranged from 16.1/100py to 41.8/100py [31-33]. The same studies reported significantly lower rates for HIV seroconversion over the same time of observation: 0.8/100py to 7.2/100py, likely due to the difference in baseline prevalence [31-33]. The greater transmissibility of HCV through percutaneous blood exposures compared to HIV suggests that even a single instance of sharing injection equipment or accidental intrafamilial transmission by sharing razors or needles for medicine could be sufficient [34]. This circumstance might have been overlooked in our study as such data has not been collected.

Relatively limited education and intervention has been targeted at the IDU community in Georgia. Primary prevention of IDU in the country has been implemented on a small scale by Non-Governmental Organizations (NGO) as fragmentary programs. Although harm reduction interventions (syringe exchange and substitution therapy) are carried out on a more systematic scale, the current coverage does not meet existing demand [35]. More regular, coordinated prevention interventions at the school, community and family level are needed. An important component may be educating women about household exposures to HIV and HCV.

As with all studies, caveats require mention. First, the study was based on chart review and data collected by physicians as part of clinical intake interviews. While all physicians at the IDACIRC are trained to work with risk groups, some patients may not feel comfortable in revealing IDU [36,37]. Second, injection-related exposure is associated with deeply ingrained cultural attitudes about IDU that highly stigmatize women, potentially increasing the likelihood of non-disclosure of IDU, especially if use is occasional. Third, measurement of exposures to HCV through medical and dental care was nonspecific. However, the relatively small risk related to medical care cannot explain the HCV distribution seen. Fourth, while majority of women with multiple sexual partners were divorced, partner information was available only on the last regular partner. Finally, 21 women were missing data either on their own HCV status or partner-related information, and were excluded from analysis.

An explosive increase in the size of the IDU population in NIS has placed drug abusers at the core of the HIV epidemic in the region, as well as a parallel HCV epidemic [4,38]. It is estimated that more than 1% of the population in Eastern Europe abuses injection opiates [39]. Our study suggests that the HIV epidemic in Georgia remains largely concentrated around the IDU community. Of the almost 80,000 pregnant women screened for HIV in Georgia, 32 women were HIV infected,[40,41] 66% of whom had a documented connection with the IDU community. Given the potential for undocumented IDU, some of the remaining 11 women also may be connected to this high-risk community or represent further transmission of HIV into the general community. There is a clear need for evidence-based interventions targeting persons with IDU and their partners, especially educational activities for young women. Mercifully, Georgia still has the chance to halt the spread of the HIV epidemic.

## List of Abbreviations

HIV: Human Immunodeficiency Virus; HCV: Hepatitis C Virus; IDU: Injection Drug Use; NIS: Newly Independent States; IDACIRC: Infectious Diseases, AIDS and Clinical Immunology Research Center.

## Competing interests

The authors declare that they have no competing interests.

## Authors' contributions

NC contributed to study design, data collection, statistical analysis, interpretation of the data, and drafted the manuscript. LAM contributed to the design, statistical analysis, interpretation of the data and provided substantial input to the intellectual content of the manuscript, PFS contributed to study design and provided revisions of the manuscript. TT contributed to study design, drafting of manuscript and provided valuable administrative support. All authors approved the final manuscript.
